# Dissociation in decision bias mechanism between probabilistic information and previous decision

**DOI:** 10.3389/fnhum.2015.00261

**Published:** 2015-05-07

**Authors:** Yoshiyuki Kaneko, Katsuyuki Sakai

**Affiliations:** ^1^Department of Cognitive Neuroscience, Graduate School of Medicine, The University of TokyoTokyo, Tokyo, Japan; ^2^Department of Psychiatry, School of Medicine, Nihon UniversityTokyo, Tokyo, Japan; ^3^Brain Science Institute, Tamagawa UniversityTokyo, Tokyo, Japan

**Keywords:** sensory detection, probability cue, decision history, functional magnetic resonance imaging, signal detection theory, prefrontal cortex

## Abstract

Target detection performance is known to be influenced by events in the previous trials. It has not been clear, however, whether this bias effect is due to the previous sensory stimulus, motor response, or decision. Also it remains open whether or not the previous trial effect emerges via the same mechanism as the effect of knowledge about the target probability. In the present study, we asked normal human subjects to make a decision about the presence or absence of a visual target. We presented a pre-cue indicating the target probability before the stimulus, and also a decision-response mapping cue after the stimulus so as to tease apart the effect of decision from that of motor response. We found that the target detection performance was significantly affected by the probability cue in the current trial and also by the decision in the previous trial. While the information about the target probability modulated the decision criteria, the previous decision modulated the sensitivity to target-relevant sensory signals (d-prime). Using functional magnetic resonance imaging (fMRI), we also found that activation in the left intraparietal sulcus (IPS) was decreased when the probability cue indicated a high probability of the target. By contrast, activation in the right inferior frontal gyrus (IFG) was increased when the subjects made a target-present decision in the previous trial, but this change was observed specifically when the target was present in the current trial. Activation in these regions was associated with individual-difference in the decision computation parameters. We argue that the previous decision biases the target detection performance by modulating the processing of target-selective information, and this mechanism is distinct from modulation of decision criteria due to expectation of a target.

## Introduction

Our decision about a sensory stimulus is known to be biased by the probabilistic information about the stimulus. Experimentally, the information can be given by presenting a pre-cue explicitly indicating the probability about which stimulus would appear on that trial (Forstmann et al., 2010; Rahnev et al., 2011; Mulder et al., 2012; Rao et al., 2012; Wyart et al., 2012; d’Acremont et al., 2013). The probabilistic information can also be obtained through experience of a sufficient number of trials (Carterette et al., 1965; Biederman and Zachary, 1970; Carpenter and Williams, 1995; Platt and Glimcher, 1999; den Ouden et al., 2010; Domenech and Dreher, 2010; Hanks et al., 2011; Turner et al., 2011). The expectation or knowledge about the likelihood of a given stimulus is shown to bias the decision performance through modulation of the distance to a decision criteria (Carpenter and Williams, 1995; Domenech and Dreher, 2010; Forstmann et al., 2010; Rahnev et al., 2011; Turner et al., 2011; Mulder et al., 2012; Rao et al., 2012; Wyart et al., 2012).

In addition to the stimulus probability, the sensory discrimination or detection performance is also shown to be biased by the subjects’ choice responses in the preceding trials (Verplanck et al., 1952; Bertelson, 1965; Treisman and Williams, 1984; Maloney et al., 2005; Gold et al., 2008; Liston and Stone, 2008; Bode et al., 2012). The previous choice has been shown to bias the subsequent performance by changing the distance to the decision threshold as in the effect of probabilistic information (Treisman and Williams, 1984; Maloney et al., 2005; Gold et al., 2008; Bode et al., 2012). The mechanism of the biasing effect of the previous choice remains open, however, because in a conventional sensory discrimination task, a stimulus presented, a decision made about the stimulus, and a motor response associated with the decision have one-to-one correspondence, and it is not clear if the previous choice effect is due to the previous stimulus, decision or motor response. Also there are studies suggesting that the previous choice affects the processing of sensory information (Treisman and Williams, 1984; Fecteau et al., 2004; Liston and Stone, 2008), and thus it remains open whether or not the previous choice effect is mediated by the same mechanism as the effect of knowledge about the target probability.

In the present study, we used a target detection task with a pre-cue indicating the probability of the presence of a target. We also presented a decision-response mapping cue after the stimulus and changed the relationship between a decision and response from trial to trial. The procedure allowed us to tease apart the effects of stimulus, decision, and response histories, and also allowed us to examine the effects of probability cue and trial history separately. Based on the signal detection theory (Green and Swets, 1966; MacMillan and Creelman, 2005) and brain activation data, we argue for distinct mechanisms of decision bias due to a decision in the previous trial and prior knowledge about the target probability.

## Materials and Methods

### Subjects

Nineteen normal, right-handed volunteers (10 females; age: 21–44) participated in the experiments. All subjects gave written informed consent to participate in this study. The study was approved by the ethics committees of the Graduate School of Medicine, the University of Tokyo and the Brain Science Institute, Tamagawa University. The data from three subjects were discarded because of excessive head movements during the functional magnetic resonance imaging (fMRI) experiment.

### Behavioral Task

**Figure 1:**
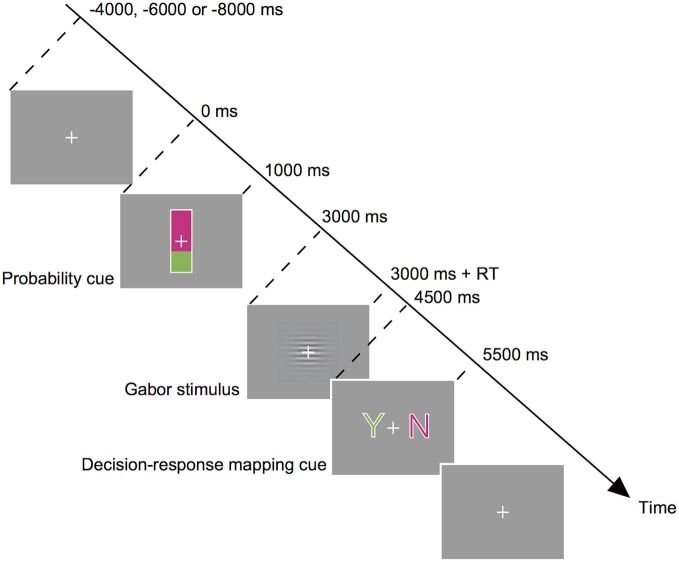
**Behavioral task**. Subjects made a decision about the presence or absence of downward motion of sine-wave gratings (target) in a noisy background. The probability of the presence of a target was informed by the height of the green bar in a probability cue (Cue-High (66.7%) or Cue-Low (33.3%)) which was presented before a stimulus. Subjects then observed a Gabor stimulus and reported completion of their internal decision by pressing two buttons simultaneously. Then a decision-response mapping cue appeared, and subjects reported their decision by pressing one of the two buttons (Y for target-present decision, and *N* for target-absent decision).

Subjects performed a visual detection task for a specific direction of motion (Figure 1). A trial started with a probability cue presented at the center of the screen. The cue was a rectangle (0.6 × 3.0°) with its bottom colored in green and its top colored in magenta. The percentage of the green shading relative to the entire rectangle indicated the probability that a motion stimulus would appear on that trial. We used two types of cue, Cue-High and Cue-Low, which indicated the target probability of 67 and 33%, respectively. On half of the trials, Cue-High was presented, and on the other half Cue-Low was presented. The actual probability of target presentation accorded with the cue. Subjects were explicitly instructed to make use of the probability cue when making a decision. Subjects were also explicitly indicated the actual correspondence between the cue and the target probability. The probability cue was presented for 1 s, followed by a blank screen of 2 s. Subjects were then shown a horizontally oriented Gabor stimulus (a sine wave grating of 1.33 cycles/degree of visual angle, enclosed within a Gaussian envelope with a full width at half maximum of 2.3 degree) embedded in white noise. On half of the trials, the sine wave grating moved downwards at a speed of 5.0 degree/s (Target (+)), and on the other half the grating did not move (Target (−)). The target presentation was also in accordance with the type of cue; the probability of target presence was 67% on trials after presentation of Cue-High, and the probability of target presence was 33% on trials after presentation of Cue-Low. Subjects indicated the decision in a two-step procedure: Subjects first reported the completion of the decision making by pressing the two buttons simultaneously using both the index and middle fingers of the right hand. This report had to be made within 1.5 s of the Gabor stimulus onset, upon which the stimulus disappeared. Reaction time was defined as the time between appearance of the grating and the simultaneous press of two buttons for reporting the completion of the decision making. Then at 1.5 s after the onset of the Gabor stimulus, a decision-response mapping cue appeared and subjects reported their decision about the presence or absence of motion by pressing one of the two buttons. The mapping cue consisted of a green letter “Y” (indicating “yes”) and a magenta letter “N” (indicating “no”) presented on either side of the central crosshair. The mapping cue remained on the screen for 1 s, and upon its disappearance subjects pressed a button on the side of a letter Y when they thought that the target was present (Gabor stimulus moving downward; Decision (+)), and pressed a button on the side of a letter N when they thought that the target was absent (Gabor stimulus not moving; Decision (−)). They used the index finger of the right hand for the right-side button press, and used the middle finger for the left-side button press. The letters “Y” and “N” can be presented on the left and right, respectively, or* vice versa*, and this letter-position relationship changed from trial to trial in a pseudorandom manner: Thus the subject used either the right index or middle finger for reporting the same decision on roughly 50% of the probability. This procedure allowed us to dissociate the decision making from motor preparation and execution processes. It also allowed us to dissociate the bias effect due to the previous decision from the bias effect due to previous motor response. No performance feedback was given on each trial, but at the end of an experimental session the correct rate of the decisions in that session was presented on the screen.

Each session consisted of 49 trials. An interval between the second button press (report on the presence or absence of motion) in a given trial and the onset of the probability cue for the next trial was randomly chosen from 4, 6, and 8 s and one session lasted for about 9 min. Throughout the session, subjects were instructed to fixate a white crosshair presented at the center of the screen. Subjects took part in eight sessions.

To determine the trial order in each session, trials with a high or low probability cue (Cue-High and Cue-Low) and trials with or without a target motion stimulus (Target (+) and Target (−)) were given pseudo-randomly within each session such that each trial type appeared equally often. Also, the order of trial types within each session was determined such that each combination of the probability cue and target type was preceded equally often by trials with high and low probability cue and also equally often by trials with and without a target.

Before the fMRI experiment, each subject took part in 3–4 training sessions, 60 trials for each session. During the training sessions except for the last one, subjects were given feedback about the accuracy of their performance on a trial-by-trial basis. In the last training session we used a two-up, one-down staircase procedure (Levitt, 1971) to determine the optimal contrast of the Gabor stimulus for each subject. The contrast was chosen for each subject so as to yield a correct rate of roughly 70%.

### Behavioral Data Analysis

We first identified the factors in the current and previous trials that influenced the target detection performance. We performed a multilevel binary logistic regression analysis with decision type (Decision (+) and (−)) as a dependent variable. As the first-level predictors, we included cue type in the previous trial (prev-Cue-High and -Low), target type in the previous trial (prev-Target (+) and (−)), previous decision (prev-Decision (+) and (−)), as well as current cue type (Cue-High and -Low) and current target type (Target (+) and (−)). Subject factor was included as the second-level predictor. Factors that significantly affected the decision were identified using backward elimination procedure based on the quasi-likelihood under the independance model information criterion (Pan, 2001), which is a refined version of Akaike’s information criterion. Removal testing was based on the probability of the Wald statistic (Bursac et al., 2008). The analysis revealed that previous decision (prev-Decision), current cue (Cue), and current target (Target) significantly affected the decision on the current trial.

We then conducted a three-way repeated-measures ANOVA on the probability of reporting the target presence, with Target, Cue and prev-Decision as within-subject factors. We also examined the effect of target, probability cue and previous decision on the time taken to make a decision. The decision time was defined as the difference between the onset of a Gabor stimulus and simultaneous press of two buttons for reporting the completion of a decision.

### Analysis Based on Signal Detection Theory

We next used the signal detection theory and examined how the probabilistic cue and previous decision affected the target detection process. For each combination of Cue (High and Low) and prev-Decision ((+) and (−)), we calculated an index of sensitivity *d*′ and that of decision criteria *c* according to the following equations (Green and Swets, 1966; MacMillan and Creelman, 2005).

$$d^{\prime }=z\left(\text{HR}\right)-z\left(\text{FAR}\right),$$

c=−(z(HR)+z(FAR))/2,

where *z* indicates an inverse of the cumulative normal distribution, and HR and FAR indicate the hit rate and false alarm rate, respectively. We conducted two-way repeated measures ANOVA with factors of Cue and prev-Decision, separately for *d*′ and *c*.

### fMRI Data Acquisition

Imaging was performed using a three tesla scanner (MAGNETOM Trio A Tim; Siemens, Germany). The functional images of 310 volumes sensitive to blood oxygen level-dependent (BOLD) contrasts were acquired in each session by T2*-weighted echo planar imaging (repetition time (TR), 2.1 s; echo time (TE), 25 ms; in-plane resolution of 3 mm in 64 × 64 matrix; 35 slices; slice thickness of 3 mm; interslice gap 1 mm). The onset of each trial relative to the preceding image acquisition was jittered in steps of 500 ms within 1 TR (2.1 s). High-resolution structural T1-weighted magnetization-prepared rapid-acquisition gradient echo images (TR, 2.0 s; TE, 1.97 ms; inversion time, 900 ms; voxel size of 1 × 1 × 1.5 mm; 192 slices) were also acquired for all subjects.

### fMRI Data Preprocessing

We used SPM8^1^ for preprocessing and analysis of the image data. The first five volumes of the images in each session were discarded to allow for T1 equilibration. The remaining image volumes were realigned to the first image, corrected for differences in slice acquisition timing, and normalized to the Montreal Neurological Institute (MNI) reference brain using a 12-parameter affine transformation along with a nonlinear transformation using cosine basis functions. The images were resampled into 2 mm cubic voxels and spatially smoothed with a Gaussian kernel (8 mm, full-width at half maximum).

The preprocessed image data were analyzed using a general linear model (GLM). For each combination of decision type (Decision (+) and (−)), target type (Target (+) and (−)), probability cue type (Cue-High or -Low), and previous decision type (prev-Decision (+) and (−)), transient activation during presentation of a Gabor stimulus was modeled as a mini-epoch with its onset time-locked to the onset of the stimulus and with its duration matched to the length of the stimulus presentation. We confirmed that all sixteen regressors used for modeling activation in this period were orthogonal each other. Transient activation during presentation of a probability cue was also modeled as a mini-epoch with its onset time-locked to the presentation of a probability cue and with its duration matched to the length of the cue presentation. The GLM also included a covariate for transient activation in response to presentation of the finger mapping cue for all trial types. Trials with no response to either a Gabor stimulus or decision-response mapping cue were modeled separately but conjointly for all the conditions as covariates of no interest. Activation during trials with no response was modeled as an epoch with its onset time-locked to the presentation of the probability cue and with duration matched to the length of trial. Head-motion parameters in six dimensions were also included in the model to remove head motion artifacts. All epochs and events were convolved with a canonical hemodynamic response function. The data were high-pass filtered with a cutoff frequency of 0.01 Hz.

### Identification of Brain Regions Associated With Decision Bias

We first tried to identify the regions in which activation during presentation of a probability cue was influenced by the cue type, but no region showed significant modulation of activation. Thus our analysis was focused on activation during presentation of a Gabor stimulus. We were specifically interested in the effects of Cue and prev-Decision, and their interactions with Target. Statistical parametric maps of t-statistics were calculated for condition-specific effects within the GLM. Images of parameter estimates for the contrast of interest were created for each subject (first-level analysis) and were then entered into a second-level analysis using a one-sample *t*-test across the 16 subjects. Statistical inferences were made based on a peak threshold of *p* < 0.001 uncorrected at a voxel level and a cluster-size threshold of *p* < 0.05 corrected for the whole brain. We used Monte Carlo simulation to determine the number of contiguous voxels to achieve the corrected significance level (Forman et al., 1995; Slotnick et al., 2003). Simulations were performed for 1, 000 times, and the minimum cluster size that yielded *p* < 0.05 corrected was 11 contiguous voxels.

For the activation of the regions identified above, we conducted ANOVA with factors of prev-Decision, Cue, and Target. To this end, for each subject, the peak activation was searched within the spherical region with a radius of 8 mm centered at the group-level peak identified above. A percent signal change relative to the inter-trial interval was calculated separately for each of the prev-Decision, Cue, and Target types.

### Correlation of Activation With Decision Computation Parameters

We then examined whether or not the activation in regions associated with Cue and prev-Decision covaried with inter-individual difference in *c* and *d*′. Based on the behavioral data, we found that *c* was significantly higher for Cue-Low than on Cue-High trials. We considered that the regions associated with regulation of *c* should show an increase in activation on Cue-Low than on Cue-High trials, and that subjects who show a larger change in *c* depending on Cue types should show a larger increase in activation on Cue -Low than on Cue-High trials. Thus the effect size of Cue on regional activation (differential activation (Diff act)), and the difference in *c* value between Cue-Low trials and Cue-High trials (differential decision criteria (Diff *c*)) were calculated for individual subject. The linear correlation between Diff act and Diff *c* was tested using the Pearson’s R correlation test.

We also found that *d*′ was significantly higher for prev-Decision (+) than on prev-Decision (−) trials. We considered that the regions associated with regulation of *d*′ should show an increase in activation on prev-Decision (+) than on prev-Decision (−) trials. We tested the significance of linear correlation between the effect size of prev-Decision on regional activation (differential activation (Diff act)) and the difference in *d*′ value between prev-Decision (+) and (−) trials (differential *d*-prime (Diff *d*′)).

## Results

### Effect of Probabilistic Cue and Previous Decision on Behavior

We conducted a multilevel logistic regression analysis in order to identify the factors in the previous and current trials that affect sensory detection performance. We first fitted the full model to the behavioral data with factors of prev-Cue, prev-Target, prev-Decision, Cue, and Target, and then tested the change in model fitting after removal of each factor. We found that removal of either prev-Cue or prev-Target factor did not significantly decrease the fitting of the model (prev-Cue: Wald *χ*^2^ = 19.6; *p* = 0.239; prev-Target: Wald *χ*^2^ = 18.7, *p* = 0.282), indicating that these factors do not contribute to the detection performance. By contrast, removal of either the prev-Decision, Cue, or Target factor significantly decreased the fitting of the model (Wald *χ*^2^ = 452.2, 236.4, and 36.3; *p* < 0.001, < 0.001, = 0.003, respectively). Thus Cue, Target, and prev-Decision are the main factors that contribute to the target detection performance. We also tested a model in which cue, target, and decision in the second trial before the current one (*n-2*th trial) were added, but addition of these factors did not improve the fitting significantly.

**Figure 2:**
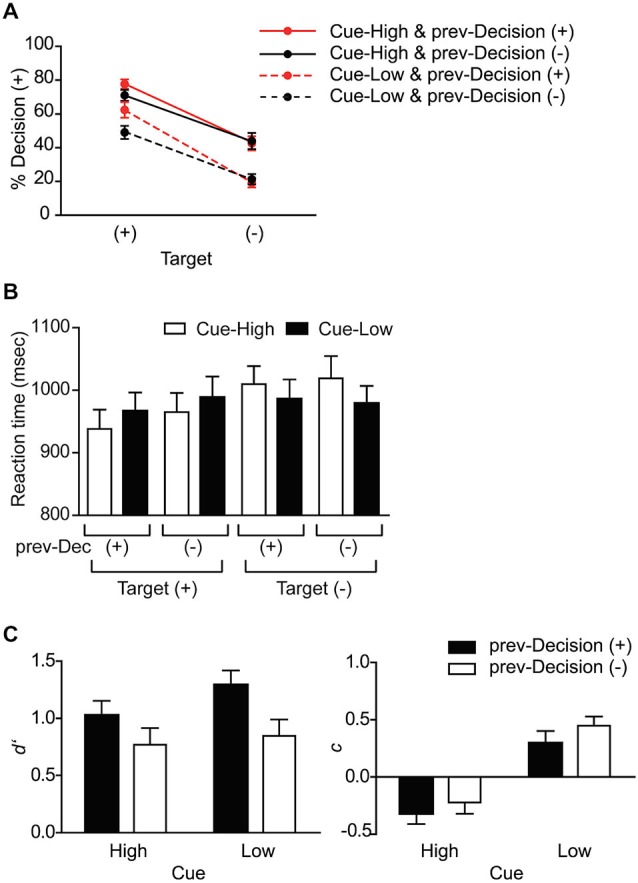
**Behavioral data. (A)** Probability of making a target-present decision (% Decision (+)). The data are shown separately for Target (+) and (−) trials, and for each Cue and prev-Decision type. Mean and SE across subjects (*n* = 16). **(B)** Reaction time. The data are shown separately for the eight conditions based on Cue, Target, and prev-Decision (prev-Dec) type. Mean and SE across subjects (*n* = 16). **(C)** Sensitivity index *d*′ and criterion index *c*. The data are shown separately for the four conditions based on Cue and prev-Decision types. Mean and SE across subjects (*n* = 16).

We then examined the effect of Target (+ or), Cue (High or Low), and prev-Decision (+ or) on the probability of making the “target present” decision (Decision (+) probability). As expected, there was a significant main effect of Target (*F*_(1,15)_ = 97.4, *p* < 0.001): The Decision (+) probability was significantly larger on Target (+) trials than on Target (−) trials (Figure 2A). The main effect of Cue was also significant (*F*_(1,15)_ = 35.4, *p* < 0.001): The Decision (+) probability was significantly larger on Cue-High trials than on Cue-Low trials, indicating that the subjects made use of the Cue information. The interaction between Cue and Target, however, was not significant (*F*_(1,15)_ = 1.73, *p* = 0.208), suggesting that the effect of probability cue does not depend on the presence or absence of Target.

Concerning the effect of Prev-Decision, by contrast, there was only a non-significant trend for the main effect of Prev-Decision (*F*_(1,15)_ = 3.39, *p* = 0.086). Unlike the effect of Cue, the interaction between Prev-Decision and Target was significant (*F*_(1,15)_ = 13.7, *p* = 0.002): On Target (+) trials, Decision (+) probability was larger in prev-Decision (+) trials than in prev-Decision (−) trials (*p* = 0.003), whereas on Target (−) trials, prev-Decision did not affect the Decision (+) probability significantly (*p* = 0.521). The result suggests that unlike probabilistic cue, the decision on the previous trial biases the decision process only when the target is present on the current trial. The interaction between Cue and prev-Decision was not significant (*F*_(1,15)_ = 0.523, *p* = 0.481), suggesting that the two factors may affect the decision process through different mechanisms. The three way interaction between Target, Cue and prev-Decision was not significant (*F*_(1,15)_ = 0.777, *p* = 0.392).

We also examined the effect of Target, Cue, and prev-Decision on the RT (Figure 2B). There was significant interaction between Target and Cue (*F*_(1,15)_ = 9.36, *p* = 0.008), without significant main effect of Target and Cue (*F*_(1,15)_ = 3.30, *p* = 0.089; and *F*_(1,15)_ = 1.05, *p* = 0.322). When target was present, the RT was smaller when Cue indicated high probability of target presence than when it indicated low probability, whereas when target was absent, the RT was larger when Cue indicated high probability of target presence than when it indicated low probability. The result is consistent with the idea that an increase in RT reflects conflict between the probabilistic information indicated by the cue and actual target presence. Concerning the decision on the previous trial, neither the main effect of prev-Decision nor its interaction with Target was significant (*F*_(1,15)_ = 0.0504, *p* = 0.489; and *F*_(1,15)_ = 1.90, *p* = 0.188).

### Effect on Decision Computation Parameters

We used signal detection theory to quantify the effects of Cue and prev-Decision on subjects’ perceptual decisions (Green and Swets, 1966; MacMillan and Creelman, 2005). For each subject, *d*′ was calculated as an index of target sensitivity and *c* was calculated as an index of detection criterion. With respect to *d*′ we found a significant main effect of prev-Decision (*F*_(1,15)_ = 15.7, *p* = 0.001) but neither the main effect of Cue (*F*_(1,15)_ = 2.75, *p* = 0.118) nor an interaction effect between Cue and prev-Decision (*F*_(1,15)_ = 0.611, *p* = 0.446) was significant (Figure 2C left). *d*′ was significantly larger when subjects reported that the target was present on the previous trial (prev-Decision (+)) than when they did not. Concerning *c* on the other hand, we found a significant main effect of Cue (*F*_(1,15)_ = 35.4, *p* < 0.001), but neither the main effect of prev-Decision (*F*_(1,15)_ = 2.62, *p* = 0.126) nor an interaction effect between Cue and prev-Decision (*F*_(1,15)_ = 0.211, *p* = 0.653) was significant (Figure 2C right). *C* was significantly larger when the cue on the current trial indicated low probability of target presence (Cue-Low) than when it indicated high probability (Cue-High). The result also suggests a difference in the mechanism by which Cue and prev-Decision affect the decision making process.

### Effect of Probabilistic Information and Previous Decision on Regional Activation

**Figure 3:**
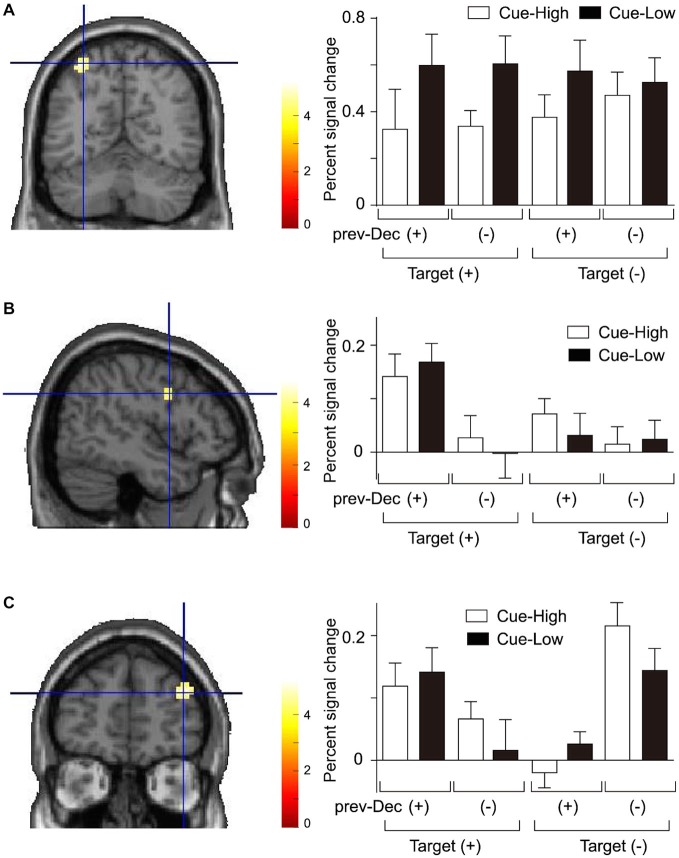
**Activation during presentation of the Gabor stimulus. (A)** Activation in the left intraparietal sulcus (IPS) showing a significant main effect of Cue. Left: The mean activation of the group-mean-peak-voxel is indicated by a cross-hair (MNI coordinate: −33, −61, 58). Right: The percent signal change is plotted for each Cue, Target, and prev-Decision (prev-Dec) type. Mean and SE across subjects (*n* = 16). **(B)** Activation in the right inferior frontal gyrus (IFG) (peak MNI coordinate: 48, 2, 28) showing a significant main effect of prev-Decision. The format is the same as **(A). (C)** Activation in the right middle frontal gyrus (MFG) (peak MNI coordinate: 39, 50, 25) showing a significant interaction between prev-Decision and Target. The format is the same as **(A)**.

According to the behavioral data showing significant main effect of Cue on the decision process, we first identified regions in which activation differed between Cue-Low and Cue-High trials. We found that a region in the left intraparietal sulcus (IPS) was significantly more active on Cue-Low than on Cue-High trials (Figure 3A, Table 1). On the activity in this region, both the main effect of Prev-Decision and that of Target were not significant (*F*_(1,15)_ = 0.076, *p* = 0.786; *F*_(1,15)_ = 0.116, *p* = 0.739). The Cue effect did not interact either with the effect of prev-Decision or that of Target (*F*_(1,15)_ = 0.517, *p* = 0.483; *F*_(1,15)_ = 3.519, *p* = 0.080).

**Table 1:** **Areas with activation during presentation of the Gabor stimulus**.

Area	Side	Coordinates	*T*-value	Cluster size (voxels)
**Main effect of Cue**
*IPS	Left	−33, −61, 58	4.40	12
**Main effect of prev-Decision**
*IFG	Right	48, 2, 28	6.79	42
IPS	Right	27, −58, 40	6.11	24
vOC	Right	36, −76, −8	4.98	13
**Interaction between prev-Decision and Target**
*MFG	Right	39, 50, 25	5.09	22
POp	Right	60, −13, 13	4.25	13

IPS: intraparietal slcus, IFG: inferior frontal gyrus, vOC: ventral occipital cortex, MFG: middle frontal gyrus, POp: parietal operculum.*regions shown in Figure 3.

We next examined the effect of prev-Decision. We found that a region around the right inferior frontal gyrus (IFG) was significantly more active on prev-Decision (+) trials than on prev-Decision (−) trials (Figure 3B, Table 1). Region-of-interest analysis additionally showed that there was also a significant main effect of Target (*F*_(1,15)_ = 12.16, *p* = 0.003). The main effect of Cue, on the other hand, was not significant (*F*_(1,15)_ = 0.188, *p* = 0.671). The interaction between Prev-Decision and Target was significant (*F*_(1,15)_ = 15.54, *p* = 0.001) with larger activity on Target (+) than on Target (−) trials for prev-Decision (+) (simple main effect, *F*_(1,15)_ = 40.01, *p* = 0.001), but no difference between Target (+) and Target (−) trials for prev-Decision (−) (simple main effect, *F*_(1,15)_ = 0.10, *p* = 0.760). The interaction between Cue and prev-Decision, and that of Cue and Target were not significant (*F*_(1,15)_ = 0.007, *p* = 0.934; *F*_(1,15)_ = 0.279, *p* = 0.605). Other regions showing a significant Prev-Decision effect were the right IPS and ventral occipital cortex (vOC; Table 1). Regions around the right IPS and vOC were significantly more active on prev-Decision (+) trials than on prev-Decision (−) trials. Region-of-interest analysis, on the other hand, showed that there was no significant effect of Target (right IPS, *F*_(1,15)_ = 0.472, *p* = 0.503; vOC, *F*_(1,15)_ = 0.077, *p* = 0.786) nor Cue (right IPS, *F*_(1,15)_ = 0.585, *p* = 0.456; vOC, *F*_(1,15)_ = 2.149, *p* = 0.163). The interaction between Cue and prev-Decision (right IPS, *F*_(1,15)_ = 1.052, *p* = 0.321; vOC, *F*_(1,15)_ = 0.105, *p* = 0.750), and that of Cue and Target (right IPS, *F*_(1,15)_ = 0.077, *p* = 0.785; vOC, *F*_(1,15)_ = 2.163, *p* = 0.162) were also insignificant.

In addition, we found that a region in the right middle frontal gyrus (MFG) showed a significant interaction between Prev-Decision and Target (Figure 3C, Table 1). For the activity at the individual peak in this region, however, there was no significant main effect of Prev-Decision nor that of Target (*F*_(1,15)_ = 3.452, *p* = 0.083; *F*_(1,15)_ = 0.073, *p* = 0.791). The Cue effect in this region was not significant, either (*F*_(1,15)_ = 1.098, *p* = 0.311). Interaction between prev-Decision and Target was also found in a region in the right parietal operculum (Table 1). For the activity at the individual peak in this region, there was a significant main effect of Prev-Decision (*F*_(1,15)_ = 5.655, *p* = 0.031). On the other hand, there was no significant main effect of Target nor that of Cue (*F*_(1,15)_ = 0.792, *p* = 0.388; *F*_(1,15)_ = 0.371, *p* = 0.552).

### Across-Subject Correlation Between Decision Parameter and Brain Activation

**Figure 4:**
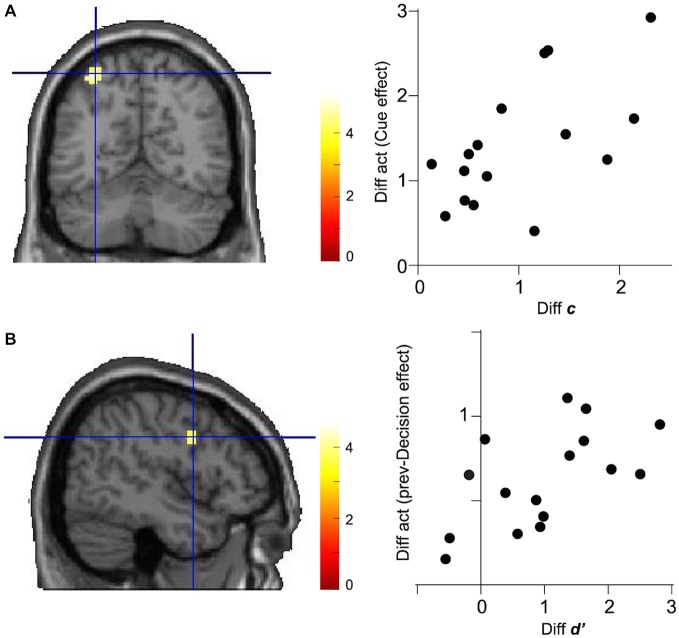
**Correlations between decision parameter and brain activation. (A)** Differential activation (Diff act) in the left IPS (MNI coordinate: −33, −61, 58) between Cue-Low and Cue-High trials is plotted against the differential decision criteria (Diff *c*). Data are shown for 16 subjects. **(B)** Differential activation in the right IFG (MNI coordinate: 48, 2, 28) between prev-Decision (+) and prev-Decision (−) trials is plotted against the difference in decision sensitivity (Diff *d*′).

We next examined whether or not the activation in regions identified in the previous section was modulated according to the change in decision computation parameters. To this end, we tested the significance of linear correlation between the effect size of Cue on the left IPS activation and the difference in decision criteria, *c*, between Cue-Low and Cue-High trials across 16 subjects. We found significant correlation between the differential IPS activation and differential decision criteria (Pearson’s correlation coefficient: 0.601, *p* = 0.014, *N* = 16; Figure 4A).

Concerning the association between the right IFG activation, on the other hand, there was significant correlation between the effect size of prev-Decision and the difference in decision sensitivity, *d*′, between Prev-Decision (+) and Prev-Decision (−) trials (Pearson’s correlation coefficient: 0.590, *p* = 0.016, *N* = 16; Figure 4B).

## Discussion

We have compared the effect of prior information about target probability and that of previous trial on a visual detection performance. We have also decomposed the previous trial effect into stimulus, decision and response factors. We found that at least within the context of target detection for an ambiguous visual stimulus, the behavior was significantly biased by the probabilistic information about the target in the current trial and decision in the previous trial. While the probability cue affected the detection performance regardless of the presence or absence of the target, the previous decision affected the performance only when the target was present on the current trial. The effects of probability cue and previous decision did not interact, suggesting independent mechanisms between the two factors. The dissociation between the probability cue effect and previous decision effect is observed in decision computation parameters and brain activation data, suggesting separate mechanisms between the two.

### Distinct Effects on Decision Performance

The mechanism of the biasing effect due to prior information about the stimulus probability has been examined fairly extensively. Regardless of whether the stimulus probability is given by an external cue or acquired through experience, the information is shown to bias the perceptual decision by modulating the decision threshold (Carpenter and Williams, 1995; Domenech and Dreher, 2010; Forstmann et al., 2010; Rahnev et al., 2011; Turner et al., 2011; Mulder et al., 2012; Rao et al., 2012). By contrast, the mechanism of the previous trial effect remains open because it has not been clear if it is the stimulus, response or decision in the previous trial that affects the performance in the current trial. In the present study, by introducing a decision-response mapping cue after the stimulus, we have been able to dissociate the effect of decision and response. Also by including cue, stimulus, and decision in the previous trial as separate factors in a multiple logistic regression model, we were able to examine the effect of each factor on target detection performance. We found that the decision in the previous trial significantly affected the target detection performance on the current trial. The cue type and the presence or absence of a target in the previous trial did not contribute to the history-dependent decision biasing effect.

We also found that the decision biasing effect differed between the probability cue and previous decision: While the effect of probability cue was observed regardless of the presence or absence of a target, the effect of previous decision interacted with the target factor. When the subjects made decision that the target was present in the previous trial, they were more likely to make the same decision in the subsequent target-present trial. By contrast, the previous decision did not affect the decision process on a target-absent trial. The results may suggest that the previous decision affects the processing of target information, whereas the knowledge about the target probability affects the decision process other than stimulus processing. This idea is supported by the analysis using the signal detection theory.

### Distinct Effects on Decision Computation Processes

According to the signal detection theory, decision computation process can be decomposed into target sensitivity (*d*′) and decision criteria (*c*) (Green and Swets, 1966; Harvey, 1992; MacMillan and Creelman, 2005). In a target detection task, the decision variable derives from the target-relevant sensory signal plus noise (Target (+) trials) or noise alone (Target (−) trials), and *d*′ corresponds to the difference in the mean decision variable between Target (+) and (−) trials. The decision variable is then compared with the decision criteria and a target-positive or -negative decision is made when the decision variable is above or below the decision criteria. The influence of the previous decision on *d*′ we have observed in the present study together with the finding that the previous decision influenced the target detection performance in Target (+) but not in Target (−) trials suggests that the decision history changes the probability distribution of the decision variable deriving from target-relevant sensory signals but not noise.

Previous studies using a sensory discrimination task have suggested that the choice history influences subsequent choices either by changing the decision criteria or starting point of sensory evidence accumulation (Treisman and Williams, 1984; Maloney et al., 2005; Gold et al., 2008; Bode et al., 2012), but there are also studies suggesting that it does so by modulating the perceptual sensitivity (Treisman and Williams, 1984; Fecteau et al., 2004; Liston and Stone, 2008). The choice history effect examined in these studies includes decision and motor response factors because there was a fixed mapping between decision category and motor effector with which to indicate the decision. Here we have removed the effect of motor response and shown that it is the previous decision that affects the target detection performance and it does so mainly by modulating the sensitivity to a target, rather than by modulating the decision criteria. Recently, Wyart et al. (2012) have shown dissociation in the effect on visual detection performance between probabilistic information about the target and task-relevance of the visual information. In that study the relevance information was shown to affect the sensitivity in information processing only for the signal present trial, similarly to the previous decision effect in the present study.

By contrast the probabilistic information was shown to modulate the decision criteria. Unlike the previous decision effect, the probability cue effect was observed for both Target (+) and (−) trials suggesting that the probabilistic information influences perceptual decision by shifting the detection criterion for both Target (+) and (−) trials to the same degree. The finding is consistent with previous studies using the integration-to-bound models (Carpenter and Williams, 1995; Domenech and Dreher, 2010; Forstmann et al., 2010; Mulder et al., 2012; Rao et al., 2012) and studies using the signal detection theory (Rahnev et al., 2011; Turner et al., 2011). In these studies modulation of the decision criteria or equivalently the starting point of the sensory evidence accumulation has been observed regardless of whether the prior information about the target probability is given by a pre-cue or obtained through experience of sufficient number of trials.

### Dissociation in the Neural Mechanism

The dissociation between the previous decision effect and probabilistic cue effect was also observed in the pattern of brain activation. The interaction between previous decision and target was observed in the MFG, and additionally in the peak activation of IFG associated with previous decision. However, the probabilistic cue did not affect the activation in these regions. Especially the differential activation in IFG depending on the previous decision was observed only in the Target (+) trials, which accords with the behavioral performance. The main effect of probabilistic cue, by contrast, was observed in the IPS, but the previous decision and target did not affect the activation in this region. The results suggest separate neural mechanisms for mediating the biasing effect between the previous decision and probabilistic information about the target. The idea is further supported by the association between activation in these regions and inter-individual difference in the degree of behavioral biasing effects. Activation in the right IFG was associated with the behavioral effect of the previous decision, whereas activation in the left IPS was associated with the behavioral effect of the probabilistic information.

Both IFG and IPS are shown to be involved in attention, but the role of each region seems to be different. While the IFG is shown to be involved in selection of internal mental representation (Nobre et al., 2004), the IPS is shown to be involved in the selection of incoming sensory information (Chun and Johnson, 2011). It could be that the previous decision about the presence or absence of a target modulates the internal representation of a target template in the IFG and thus selectively affects the performance on Target (+) trials. The probabilistic information, on the other hand, may modulate the gain of sensory information processing in the IPS. Another possibility is that the IFG is a part of the ventral attention network and is involved in stimulus-driven attention, whereas the IPS, which is a part of the dorsal attention network, is involved in endogenous top-down control of attention (Corbetta and Shulman, 2002). While the stimulus-driven attention mechanism in the IFG may be triggered by the memory of having made a specific decision, the top-down attention mechanism in the IPS may be modulated by the explicit knowledge about the likelihood of target appearance as suggested in a previous study (Wyart et al., 2012).

Findings from single unit recording studies on monkeys can also be taken to support the present finding. It has been shown that the probabilistic information changes the baseline activity of neurons in the monkey LIP region (equivalent to the human IPS) (Platt and Glimcher, 1999; Hanks et al., 2011; Rao et al., 2012), suggesting that probabilistic information modulates the starting point of the sensory evidence accumulation and effectively reduces the decision criteria. A decrease in the IPS activity during a decision period observed in the present imaging study may reflect an increase in the activity during the pre-stimulus period. In contrast to the probabilistic information, the choice history does not affect the activity of LIP neurons (Gold et al., 2008). Instead, the trial-by-trial change in the offset of decision variable, which has been shown to reflect the choice history effect, has also been shown to be correlated with responsiveness of frontal eye field (FEF) neurons as measured by oculomotor response induced by microstimulation. In the present study, activation in the IFG rather than in the FEF may reflect the fact that a hand motor response was used to indicate the decision.

Previous human imaging studies have shown that not just the IPS but a more extensive set of regions are involved in the biasing effect due to probabilistic information: The regions include dorsolateral and inferolateral prefrontal cortices, anterior cingulate cortex, premotor cortex, putamen, and hippocampus, in addition to parietal regions (den Ouden et al., 2010; Domenech and Dreher, 2010; Forstmann et al., 2010; Rahnev et al., 2011; d’Acremont et al., 2013). Concerning the effect of previous trials, there are studies in which activation in the FEF is modulated by the previous choice (Manoach et al., 2007; Akaishi et al., 2014). In the present study, we found that activation in the IFG is associated with previous decision. An interaction between the previous decision and current target is observed in the MFG as well as IFG. It could be that activation in a selective set of regions in the present study is due to the removal of confounding factors by including multiple factors in the fMRI data analysis model.

### Conceptual Framework of Decision Bias Due to Prior Information

There are different sorts of prior information such as memory and expectation of an upcoming stimulus, decision, and response, and either of them could affect the behavior on the subsequent trials. The present study has made it clear that it is the previous decision that affects the subsequent target detection performance. A recent study has also shown that the previous decision contributed more to the decision bias than the previous stimulus or response, and that this previous decision effect is significantly larger when the decision has been made on an ambiguous stimulus in the previous trial than when it was made on a salient stimulus (Akaishi et al., 2014). When a stimulus is ambiguous, the decision has to be made based on an endogenous signal, and this signal may be carried over to the subsequent trial and bias the decision.

The key finding in the present study is that the previous decision seems to affect the processing of target-relevant information. When subjects believed that the target was present in the previous trial, this belief may facilitate the processing of target-relevant sensory information and lead to a higher probability of a target-present decision. When a target is absent in the subsequent trial, no decision biasing effect is observed. This may suggest that the effect of previous decision is specific to the processing of target-relevant sensory information, and does not modulate the processing of noise. Such selective modulation seems to be mediated by the prefrontal region known to be involved in the control of selective attention (Desimone and Duncan, 1995), and the finding of IFG and MFG activation associated with previous decision effect is consistent with the idea. By contrast, the probabilistic information about the target is less selective; the biasing effect is observed regardless of the presence or absence of the target. In contrast to the biasing effect of the previous decision on on-line processing of target information, the probabilistic information seems to take effects by adjusting the decision threshold before the stimulus presentation, and thus non-selectively affects processing of both target information and noise.

The dissociation between the previous decision effect and probability cue effect may reflect the fact that while the previous decision affects the decision process through actual commitment of the decision, the probabilistic information influences the behavior by providing abstract knowledge about the event occurrence. The dissociation, however, does not necessarily indicate the independance between the two mechanisms. Our claim is that under the context of sensory detection for an ambiguous stimulus, previous decision plays a major role in biasing the subsequent decision, and it does so via a mechanism that differs from the one involved in the decision bias due to probabilistic information. The interaction between the multiple decision biasing mechanisms should be explored in future studies.

## Conflict of Interest Statement

The authors declare that the research was conducted in the absence of any commercial or financial relationships that could be construed as a potential conflict of interest.

## Footnotes

^1^http://www.fil.ion.ucl.ac.uk/spm
